# Restored Life of Elite Athletes after Spinal Cord Injury

**DOI:** 10.3390/ijerph19148441

**Published:** 2022-07-11

**Authors:** Grzegorz Zurek, Agata Goraczko, Alina Żurek, Maciej Lachowicz, Katarzyna Kujawa

**Affiliations:** 1 Department of Biostructure, Wroclaw University of Health and Sport Sciences, 51-612 Wroclaw, Poland; grzegorz.zurek@awf.wroc.pl (G.Z.); maciej.lachowicz@ideis.pl (M.L.); katarzyna.kujawa@awf.wroc.pl (K.K.); 2 Clinic of Neurorehabilitation, 54-519 Wroclaw, Poland; 3 Institute of Psychology, University of Wroclaw, 50-527 Wroclaw, Poland; alina.zurek@uwr.edu.pl

**Keywords:** spinal cord injury, elite athletes, adjustment, athletic identity, coping

## Abstract

Spinal cord injury (SCI) affects every aspect of human life: medical, psychological, social, material. People with SCI face a variety of secondary conditions (e.g., chronic pain, urinary tract infections, cognitive impairment) that place a significant emotional burden, resulting in an increased risk of depression and reduced quality of life. The purpose of this study was to better understand the coping strategies and to identify factors that promote or hinder the successful adjustment of elite athletes after SCI. Individual semi-structured interviews were conducted with eight top athletes after spinal cord injury. The interviews were recorded, transcribed, and then thematically analyzed using MAXQDA software. Thematic analysis identified the following categories: coping, athletic identity, and adjustment. The results of the study indicate that loss of functional ability does not cause loss of athlete identity. Elite athletes live a life consistent with this identity, attempting to maintain it despite the loss of physical fitness. Involvement in sports provides meaning and is a positive factor in the process of disability acceptance, which is essential in the process of adjustment to injury and also provides group belonging.

## 1. Introduction

Spinal cord injury carries a number of consequences affecting every dimension of human life: health, psychological, social, material [1]. In addition to loss of functional abilities, sensory deficits, bowel and bladder dysfunction, respiratory and circulatory dysfunction, and sexual activity occur [2]. In addition, people after SCI struggle with secondary conditions such as chronic pain (musculoskeletal, neuropathic, visceral), spasticity, urinary tract infections, cognitive impairment, chronic fatigue, and thermoregulatory disorders [2,3]. These factors represent a significant emotional burden and cause an increased risk of depression (which occurs in 22.2% of patients on average) and consequently reduced quality of life [4,5,6]. 

Most epidemiological data on spinal cord injury are extrapolations based on data collected in clinical settings [7]. The global incidence of traumatic SCI (TSCI) is estimated at 23/million people in 2007 (179,312 cases per annum worldwide) [8]. The prevalence of TSCI worldwide ranges from 236 to 1298/million population, and in addition to regional variations, there has been a trend of increasing prevalence worldwide in recent decades [9]. The highest proportion of TSCI caused by sport was in Russia (32.9%), followed by Fiji (32.0%), New Zealand (20.0%), Iceland (18.8%), France (15.8%), and Canada (13.1%) [10]. The sports that cause the greatest number of TSCIs in the most countries were: diving, skiing, rugby, horseback riding, football, cycling, and motor racing [10].

Such a traumatic injury and change from ones previous life requires adjustment to the new situation. There are several theories explaining the adjustment process after SCI: stage theory (Trieschmmann, 1980), response shift theories (Spranger and Schwartz, 1999), the transactional model of stress and coping (Lazarus and Folkman, 1984), and the SCI Adjustment Model (SCIAM; Middleton and Craig, 2008) [11,12,13,14]. The latter was developed to explain all aspects of SCI adjustment by combining different models and theories. SCIAM assumes that adaptation occurs under the influence of biological, medical, psychological, social, cultural, political, and religious factors, between which there is a synergistic relationship [15]. Pre-disease factors also have a strong influence on adjustment, which occurs through the assessment of the situation in relation to modifying factors at a given time and the use of available coping strategies. When positive stressors prevail and personal resources are high, the patient perceives the situation as positive [15]. In a study by Elfström et al. (2002), three psychometrically valid and reliable ways of coping with SCI were identified: acceptance of the physical consequences of the injury, fighting spirit including efforts to maintain independence and make the best use of life in spite of the circumstance, and social resilience, which is maladaptive [16]. Optimal adjustment is led by a process that consists of the following stages: displacement, anger, bargaining, depression and desperation, and ultimately acceptance of the new reality and posttraumatic growth [11]. As long as the negative feelings of anger, sadness, and depression are transient, they do not constitute an obstacle to long-term adjustment. The development of resilience is facilitated by social support and focus on problem-solving [15]. 

Despite the significant emotional burden in patients after SCI, previous studies indicate health long-term psychological adjustment of individuals in this group [17,18,19,20]. In a study by Bonanno et al., with the participation of 233 participants, it was shown that most patients after SCI have significant psychological resilience [18]. Tedeshi and Calhoun (1995) introduced the term posttraumatic growth (PTG), which refers to the post-trauma achievement of a number of benefits, deeper understanding of the world or oneself, and personal growth through ways of coping with difficulties [21]. The results of Byr’s (2016) study in a group of 169 individuals with paraplegia show that in terms of PTG, the highest degree of positive change was indicated in appreciation of life [22]. In this study, coping strategies such as religion, focusing on the problem, humor, and hope are 60% responsible for PTG [22]. Kalpakjian et al. studied 824 participants, with most of them experiencing PTG, and the biggest change they observed was the discovery that they were stronger than they thought [23]. This phenomenon discovered in a group of athletes with disabilities can be transferred to a group of healthy athletes finishing their careers. Smith and McManus point to the shortcomings of programs that foster positive adaptation in athletes who are ending their athletic careers and the opportunity to utilize the experience of former athletes in developing programs to help minimize stress and make appropriate lifestyle choices [24]. 

There are numerous studies on the involvement in adapted sports of individuals after SCI and the physical as well as psychosocial benefits of this, including improved quality of life, life satisfaction, better community integration, and the development of new friendships [25,26,27,28]. In a qualitative study by Hawkins et al., participants were elite badminton players [29]. In contrast, research on athletes who have sustained a spinal cord injury as a result of sport has been undertaken extremely rarely. In a study by Sparkes and Smith involving 14 male athletes who had suffered SCI during rugby games, the majority of subjects felt strong hope associated with a belief in recovery [30]. Badenhorst et al. describes the quality of life of 90 individuals with rugby-related SCI as higher than the control group [31]. 

However, to the best of the authors’ knowledge, no self-reported quality of life study has been conducted to date using interviews with world-class athletes who have suffered a spinal cord injury; therefore, the material collected is a valuable and unique source of information. The purpose of this study was to explore the life histories of the subjects and to provide a thorough analysis that allowed for a deeper and better understanding of the coping strategies they use and to identify factors that promote or impede the successful adjustment of elite athletes after SCI.

## 2. Materials and Methods

This paper deals with the life histories of eight prominent athletes who suffered spinal cord injuries. A descriptive–qualitative method was used in the study.

### 2.1. Participants

The following inclusion criteria were used: sport achievements at the minimum national level (winning a medal in at least one sporting event of national rank) before SCI, spinal cord injury (tetraplegia or paraplegia), and informed consent to participate in the study. Due to the nature of the study, participants included in the project had to know either Polish or English to a degree that allowed free communication and understanding. 

Based on the available internet sources, sports committees on spinal cord injury among elite athletes were reviewed. Consequently, 32 subjects meeting the above criteria were selected and sent invitations to participate via e-mail. Sixteen subjects responded positively to the invitation, but three did not meet the inclusion criteria and five did not return the consent to participate in the study. Sociodemographic and SCI data are presented in Table 1.

To conduct the study presented in this paper, the authors obtained the consent of the Senate Research Ethics Committee of the University School of Physical Education in Wroclaw, Poland (corresponding ethical approval code: 37/2018, art.27, Dz.U.1997, poz.553). Study participants gave informed consent both to participate in the study and to the publication of its results in accordance with the guidelines established by the Declaration of Helsinki. 

### 2.2. Study Design

**Figure 1:**
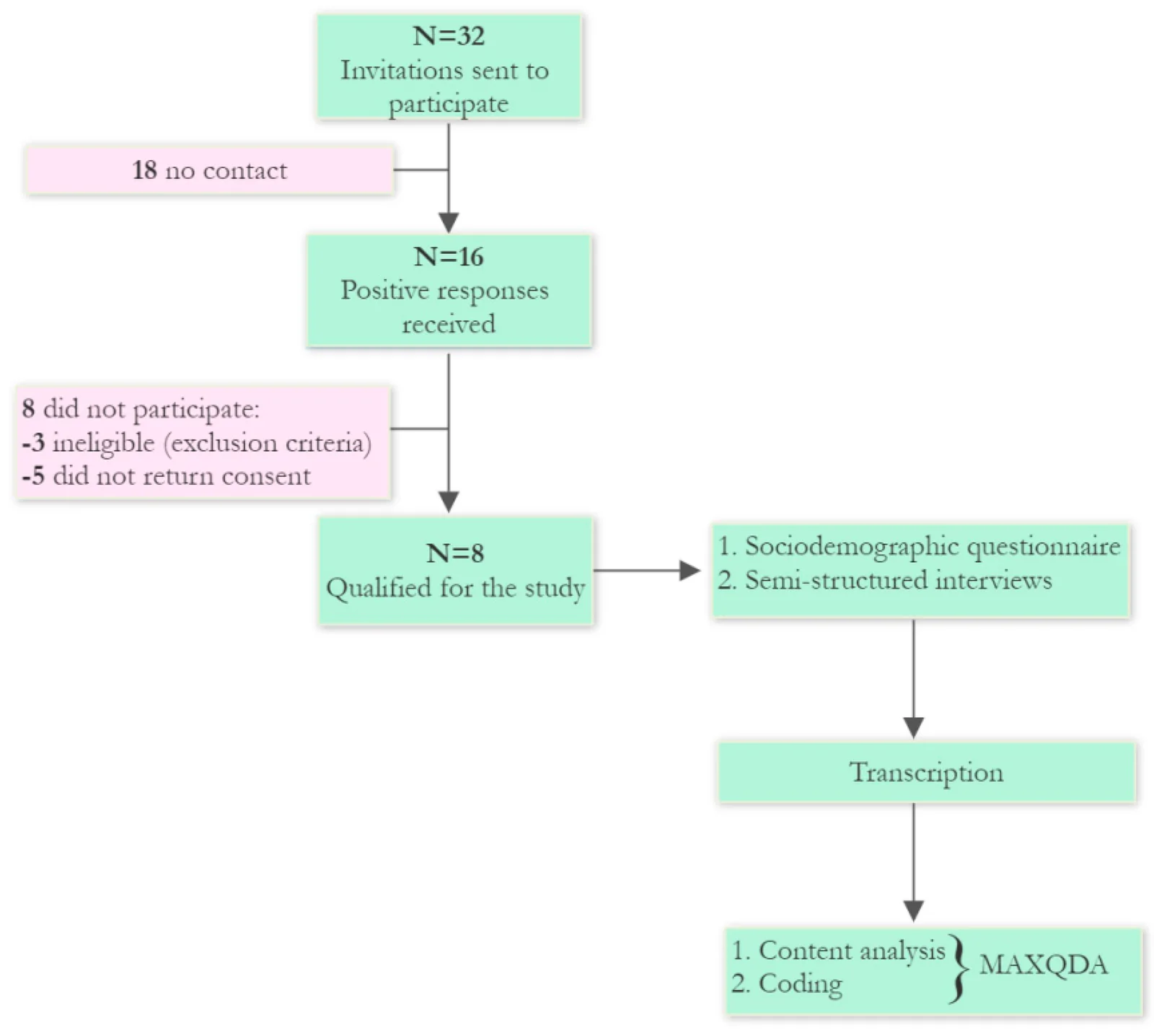
The flow chart of the study design.

The final participants in the study were elite athletes who were interviewed and returned completed sociodemographic questionnaires. Figure 1 shows the study design along with the recruitment stage of the participants.

The study used semi-structured interviews, which is the most commonly used method of qualitative research in the health care field [32]. Due to the fact that participants live in different regions of the world, the interviews were conducted via instant messenger, recorded, and then transcribed. This made it possible to conduct each interview at a time and place most convenient for the participant, which contributed to a more productive interview process. Prior to the interview, participants were given a consent form, which included a description of the project, its purpose, and rules of ethics and anonymity, which they read and verbally agreed to at the beginning of the interview. The interviews were conducted by the second author. The duration of each interview was between one and a half and two hours. The interviews consisted of key questions that helped define the areas the authors wanted to explore while allowing the interviewee to speak freely. The flexible format of interviewing allows for the discovery and development of information important to the participant [33]. Once verbal consent to participate was obtained, the interview began with a general, simple question, “Please tell us something about yourself”, in order to help the respondent feel at ease, build trust and rapport, and ultimately obtain valuable and worthwhile data addressing more sensitive and difficult topics [33].

### 2.3. Data Processing and Analysis

Data were collected in 2019–2020 from the semi-structured interviews. MAXQDA^®^ version 2022 software (Release 22.1.1, VERBI GmbH, Berlin, Germany) was used to organize the data for qualitative analysis and coding. The thematic analysis of the content of the interviews proceeded in several stages. In the initial stage, two researchers analyzed each statement by giving it a label similar to the terms used by the respondents. In this way, semantic units or codes corresponding to the main idea were extracted. In the second stage, the researchers focused on the analysis of subcategories, where units of similar meaning were organized into categories. Eventually, three main meaning categories were identified: (1) sport identity, (2) coping in the initial pattern of reaction to the accident, and (3) adaptation in the long-term pattern of behavior after the accident.

## 3. Results

Following the thematic analysis, three categories emerged: coping, sport identity, and adjustment. The themes along with the categorization and matching quotes are presented in Table 2.

### 3.1. Coping—An Early Adaptive Pattern

Participants described various ways of coping with a difficult post-accident situation. These included a variety of behaviors or defense mechanisms to protect against an existential sense of threat. Analyzing the interviews, we identified the following adaptive strategies: rationalization, repression, denial, postponing the cognitive confrontation with the consequences of the accident, analyzing/reflecting on the causes of the accident, seeking information about the chances of walking, learning about conventional and unconventional treatments, believing in God, seeking to make sense of the accident, behaviors to relieve tension such as crying and seeking contact with relatives and friends, and working. By adaptive pattern, we mean the presence of different coping strategies and the functions they perform. We observed the above strategies in all cases, although in different configurations and with varying degrees of intensity. We did not observe any recurring pattern in the order of their occurrence, co-occurrence, or intensity. They also varied over time depending on their effectiveness. Their function was to bring about emotional and cognitive equilibrium and to reduce feelings of existential anxiety.

Up to the point of SCI, the participants had experienced accidents, and there were already shorter or longer moments of interruption in their athletic careers. They were aware of the risk of accidents and did not dwell on the thought that a serious injury such as SCI could befall them, especially since it was extremely rare in the sports they practiced. An accident is a turning point in the lives of participants who have played sports professionally. P1 emphasizes how difficult a mental challenge this incident was:

*P1: It is really difficult, your mental challenge is to keep going. Everything was a challenge when I woke up.*

*P4: There were some dark days, and I really struggled initially but I had such a lot of support*

P2 was aware from the first moment of the severity of the injury and the difficulties he would face. He points to the very important words of the doctor who gave him the diagnosis.

*P2: I interrupted him ‘That means I’m paralyzed from now on. Is that right?’ and then he said ‘Yes’ that was the moment that brought some tears to my eyes. Then he continued talking immediately what was very important because he said ‘I have to remind that today I have a healthy head, a healthy mindset, and quite healthy hands, and these components make sure that I can have quite normal life. Yea and maybe this was the most important sentence in this whole journey which I’m since that day.*

In contrast, P3, in retrospect, doesn’t even remember the moment of collapse.

*P3: From the very beginning I had a positive and fighting attitude. I didn’t understand myself fully but I didn’t have a single moment of breakdown.*

P6, in the first moment after opening his eyes, felt lucky to be awake and still alive despite such a serious accident. Regardless of the shock experienced and the challenges posed by the consequences of SCI and the initial struggle, the participants were set to fight. Contrary to the diagnosis they had heard, the participants were eager to prove it wrong and were confident of a complete rescission, focusing on intensive rehabilitation.

*P7: I think in psychology it is described somehow, some kind of denial that I can do it. That 99 out of 100 couldn’t make it, but I can make it. This is related to what I said, that after the accident I spent several years on rehabilitation, that very hard, professional work will have some effect, and although history teaches us that in some cases there is no effect. I explained to myself that there was no effect because these people were not determined enough, but I explained to myself that if I spent years I would think I would succeed.*

At the same time, the hope of fully recovering allowed some to survive the most difficult initial moment:

*P5: It was good that I had the hope that I had. I was 100-percent convinced that I was going to walk out of hospital. Without this hope, it would be so much more depressing.*

### 3.2. Sports Identity

Analyzing the interviews, we noticed that the participants constructed their “self” in the area defined by the culture of the sports group they belonged to before the accident. These groups provided them with specific categories to describe themselves. Their personal identities were responses to questions about strong relationships with the sports group, parents, coaches, or athletes as important people in their lives. Membership in a particular professional group was therefore an acquired identity, formed as a result of group membership and consciously chosen. In the pre-traumatic past searches for answers to the questions Who am I? Who am I supposed to be? Who do I want to be?, our participants had constructed their sport identities:

*P8: My life has always revolved around sports because my dad was a sportsman Well, I must have been soaked in it like a shell of an egg and this sport was absorbed by me as well Well, there was still some soccer later on because there was also speedway Well, I ended up on this speedway the way I ended up, Skiing and cycling were also passions that I continue somewhere now because I also ski and cycle, so it was such a cool life because maybe in the summer I rode speedway, cycling and then came winter. Since autumn I was already skiing because I also had a ski school. Also I was teaching skiing some trips we organized, training for children for adults of all ages and such a life to envy…*

Participants after their accidents found certain advantages of having been athletes: physical strength, support from fans and sponsors, and opportunities for impacts on the lives of others:

*P2: They also knew about that I was a sportsman so I could handle some things a bit better.*

During their conversations with us, they referred to their sports past and planned for the future. They expressed the need to belong to their previous sporting environment and to identify with their immediate surroundings. Despite the fitness loss, they did not lose their identity. In accordance with their vision of themselves, they set new goals and chose ways to achieve them. They sought out activities and lived their lives in such a way as to live up to that identity even though they lacked functional capabilities. Achieving identity–life compatibility helped them to accept their disability at the same time:

*P5: My identity has always been of an athlete and so now not to have that as my identity was frightening really scary during that time. ..... I think all of these things sort of happening when they happen gaining my confidence back from tennis and basketball. You know sort of being more outgoing. Where is for a while i was such an introvert because I didn’t know I was not comfortable with myself. So you know it’s taking a long years. You know what I think it’s different for everyone in a chair. I learned to be totally ok with myself and that changed a lot for me not only in sport and to make the connections that I’ve made and to be able to talk about you know an accident and my process and my journey throughout the whole thing.*

Most of the participants took up sport activities by changing the discipline: P2 rugby and skiing, P3 wheelchair dancing, P5 wheelchair basketball, P6 race car driving and handcycling, P7 canoeing, and P8 hand-cycling.

*P7: I saw it and it made me feel really stupid, ashamed. Because I saw, let’s call it broadly, people like me, and they were lifting such weights* *…How do we count these circles? How many are there? And at that moment the athletic soul was awakened, the perversity that I also have to here. And that was the beginning.*

*P8:* *…in fact life didn’t end that you can still do sports, maybe a little bit in a different form because unfortunately for that you need some kind of special equipment, but it was possible. Well there was some kind of a signal that I was saying that he can continue to do that skiing that I used to do before the accident, I’m still doing it, and that’s how it went.*

Participants are also involved in sports life as coaches, as co-organizers of events, and as heads of foundations they started to support athletes after SCI.

*P3: I still sometimes take part in, appear at the start or finish line of, for the benefit of the earth, or marathons, also supporting runners, because I myself enjoyed running before the accident. And after the accident, from friend to friend, it so happened that I also support the runners. Also at some stops, finish line or start I am with them. I don’t run marathons with them because my arms would fall off, but at least this way I spend time with them*

### 3.3. Adjustment—A Long-Term Adaptive Pattern

Because of the long time that had passed since their accidents (5–17 years), these participants had gone through various stages of adjustment and were currently in the stage of coming to terms with the consequences of the injury and experiencing posttraumatic growth. By analyzing the interviews, we distinguished: (1) psychological resilience as evidenced by regaining meaning, (2) finding motivation to live both externally and internally, and (3) behavioral recovery-setting goals such as undertaking new sporting and advocacy activities. By long-term adaptive pattern, we mean the presence and functions of different coping strategies they had used over the years of their recovery. Their aims were primarily to maintain a state of emotional and cognitive balance, to come to terms with the consequences of the injury, and to experience posttraumatic growth in the new reality.

#### 3.3.1. Finding the Meaning of the Accident

A complete acceptance of post-accident reality is evidenced by finding meaning and positive aspects of the incident. P3 points out how much she has learned and achieved and how life in a wheelchair has opened up new perspectives and opportunities for her. P2, because of winning the court battle for compensation with the sports committee, unequivocally states: “at least now my wheelchair has a reason”. P2 and P3 have reevaluated their lives and, thanks to the accident, do not complain about small adversities. They always try to see the positive aspects of every situation. P4 finds meaning from the accident in being a support for other people:

*P4: I believe, my accident happened for a reason so I can support young people who have a similar injury to me but don’t have that support I received.*

#### 3.3.2. Sources of Motivation

For participants, sources of motivation are their families, the positive effects of the work they put into rehabilitation, and the admiration of others present in their lives:

*P1: After some time I was able to keep myself standing, it was so good for my mental condition, it was so inspiring and motivating.*

*P2: When you see other people being proud of you because you just managed to move in the wheelchair.*

Participants are also motivated by the stories of other people with disabilities who lead happy and fulfilled lives despite their disabilities.

*P3: I watch motivational speakers on youtube who are also struggling and overcoming these barriers.*

An additional motivation for both P7 and P8 are their rivals and people who make things difficult for them:

*P7: Negative motivation, I will tell you what it is, in short, it means that when someone throws obstacles under my feet and is motivated by some, I don’t know, malice, which I experienced. This motivates me very much to show them, to rub their noses in it.*

#### 3.3.3. Acceptance of Disability

At first, the participants were fully resistant and did not identify with the world of disabled people. With time, they started to realize themselves in another sport and to integrate into the society they started to belong to.

*P5: I didn’t want to be around anyone in a wheelchair because that made me really realise that was my world but when I did and it was when I started to go out to the tennis practices and I would hang out with everyone that was playing at the time. It makes it a little bit more bearable and so like. I ended up I love the fact that I’m part of this community.*

They discovered that despite their disabilities, they could continue to be happy and lead fulfilling lives:

*P2: Training, working, being outside, that wheelchair live is possible.*

*P4: For once I didn’t feel pity because of my situation. That’s amazing.*

P3 also aims to break stereotypes and make people aware that people with disabilities need full acceptance and want to live normally, without drawing unnecessary attention to themselves:

*P3: I try to show that a person in a wheelchair is not a freak but a normal person who needs normality, tenderness, and to be with other people.*

Acceptance of one’s condition is evidenced by the ability to freely tell one’s story including the circumstances of the accident to motivate both the disabled and the healthy communities with one’s attitude:

*P8: Some companies motivate employees and show them that they are complaining having actually everything having arms, legs they are there complaining that they don’t want something and this shows them that they are half the guy that is 50% and doing twice as much work as them.*

P4 and P8 have set up foundations to support and bring together post-injury athletes and share their experiences and journeys. P8 is also a lecturer in sport and tourism for people with disabilities.

#### 3.3.4. Goals

Each of the participants was able to identify specific goals they were currently setting for themselves. Some gave general goals unrelated to the accident: to be happy, to start a family, to rear children, to finish building a house. Goals directly related to the accident concerned the continuation of rehabilitation and the improvement of functional abilities:

*P2: I have reached all my milestones. In my situation now my goal is to improve my walking on crutches, or without crutches.*

At the same time, experiencing difficulties related to the wheelchair, P2 sees the need to adapt spaces to people with disabilities and to remove architectural barriers:

*P2: I want to raise the focus of the public when they build things to think about disable.*

Further objectives are to support people with disabilities by giving motivational speeches and, in the case of P4 and P8, to propagate and review the activities of the foundations they have established:

*P4: The ambitions in my life is to make a foundation full of friends and spread the word about disability. I think as well we are changing the world for many disabled people, we empower them to do their best, make the most of their lives, try to look at the positives of their lives. That is massively important in this situation. We just try to give people hope that they can move on with their life. We try to give them a purpose to wake up every morning. that’s really important.*

P5, P6, P7, and P8 changed sports but continue to play sport at a professional level, so their goals also include further achievements in this area.

## 4. Discussion

In this study, we presented the thematic analysis of the responses from eight interviews with elite athletes who sustained spinal cord injury during their sport careers. The analysis aimed to provide key information about the process of adjustment to the new post-injury reality and the factors that led to the recovery of emotional balance and a satisfying life despite the consequences of SCI.

Of the three strategies listed by Elfstrom et al. for coping with SCI in the first period immediately after the accident, participants in our study used fighting spirit [16]. It involved undertaking intensive rehabilitation and hope for restitution, which, as P5 points out, enabled her to survive the most difficult moment. On the one hand, conveying an adequate diagnosis and realistic chances for the patient are important, but on the other hand, hope should not be taken away as it is essential in the fight. In the qualitative studies by Hawkins et al. and Smith, participants’ hope was also generated from the prospects of a cure in the future [29,30]. However prolonged, unrealistic hope and lack of acceptance is a significant barrier to achieving optimal adjustment. Pollard and Kennedy indicated that something that happens immediately in the post-injury period has profound implications for adjustment later [19]. This points to the important role of intervention by health professionals as those who interact with patients directly in the pre-injury period. P2 still paid particular attention to the words of the physician despite the lapse of 5 years since the accident (see results section). In a study by van Diemen et al., participants found the motivational attitude of professionals to be stimulating, which was seen as a post-positive element of mental adjustment [34].

Erik Erikson believes that identity is a sense of being special while at the same time being integrated into a social frame of reference in which one plays a role [35]. In the literature, there is a notion of group identity, which is a way of defining oneself by belonging to different types of social groups. The common factor that characterizes all participants in this study is their athlete identities, which, irrespective of the consequences of SCI, is still the apex in the identity hierarchy. The social frame of reference for the subjects was the groups of athletes they had come from. Coming to one’s own athletic identity is a long, arduous process. The SCI that emerged, although it was a hindrance to self-realization and caused internal conflicts of tension, still constituted the identity status quo of our subjects. Constructing one’s personality and sense of self along with hopes for the future based on pre-injury life was also observed among the respondents of Zuchetto et al.’s study [35]. Sport identification can affect individuals experiencing SCI in two ways. Cases have been reported in the literature where strong athletic identity hindered or even prevented the adaptation process [36]. An extreme example is a former professional rugby player committing suicide after SCI [37]. On the other hand, athletic identity can be used as a facilitator of recovery and support positive long-term adaptation [25,26]. During the preoperative period, the participants in our study experienced shock and loss, which was compounded by the thought of losing the opportunity to play sport. As time passed and the participants slowly accepted and embraced their disability, they began to explore ways and opportunities to maintain, continue, or even enhance their sport identity, which was an important step in the adaptation process. The greatest limitation that cannot be eliminated is tetraplegia with a complete lack of functional ability to participate in even paralympic sports [38]. According to Sparkes, the experience of an injury that threatens the fulfillment of the role of athlete negatively affects the personal identity of individuals who strongly identify with that role [39]. This could suggest a serious problem without a solution for professional athletes experiencing SCI at level C5 or below. However, it is an interesting observation that P1 and P4, with high spinal cord injury above the fourth cervical vertebrae, found opportunities to pursue a sport identity. As it turns out, it can also be rewarding to passively participate in sporting events, to be a coach for a sport one previously played, to organize sporting events, and to run a foundation to support athletes who have suffered SCI. Other participants began to become involved in disability sports, and new group affiliations began to define changes in social identity, successively leading to changes in behavior. The meaning and value of membership in the new community provided an important potential for positive adherence to therapy, as has been observed in previous research [38]. In a study by Tasiemski and Brewer (2011) with 1034 individuals with SCI, amount of weekly sport participation was positively related to athletic identity, and team sport participants reported better psychological adjustment than individual sport participants [38]. In a study conducted on a group of 80 patients after SCI, participants who took part in regular physical activity had better fitness, greater independence, and better functional status [26]. Furthermore, Silveira et al. in a study of 150 males with tetraplegia noted a correlation between the frequency of sports participation and reductions in psychological distress [27]. A good predictive model for sport participation of individuals with acquired physical disabilities is the Health Action Process Approach (HAPA), which may be valuable in preparing sport promotion programs for populations with SCI [40].

Participation in sport for people with disabilities has been very helpful in their acceptance of their disabilities, which is extremely important because the lack of ability is a significant barrier that must be overcome in order to achieve positive adaptation [19,41]. Unfortunately, according to Perrier et al. (2015), only about 3% of individuals with acquired physical disabilities participate in sport, while 50% of individuals in this group expressed interest in being able to participate in adapted sport [42]. This indicates the need to promote sport in this population. The participants in our study and their stories can serve as examples for both elite athletes and other people with disabilities. It is worthwhile for health care professionals to have information on foundations supporting people with SCI in their country, as this can be important information for the patient and an important step in starting a positive adaptation to a new life situation. P4 and P7 have set up foundations that aim to facilitate the difficult process of adaptation, and the stories of elite athletes can be a valuable example that you can lead a fulfilling life despite your disability. Additionally, familiarity with the stories of elite athletes can be a helpful guide that health care professionals can use in their work with patients to build motivation, foster positive thinking, and develop the drive to be as independent as possible.

Athelstan and Crewe (1979) found that individuals who were injured as a direct result of their own behavior were better adapted than those who were injured accidentally, which applies to all participants in our study except P7 [43]. Participants in our study came to a reflection that allowed them to make sense of the accident and, further, to list the many benefits and changes in themselves that it brought. As in previous studies, participants indicate a greater appreciation of life and their health, a reduction in complaining, and the discovery that they were stronger than they thought [18,19,40].

As authors of the paper, we recognize limitations of our study, which are primarily related to the specific study group of elite athletes with SCI. The inclusion criteria used may have influenced the size of the study group, although we made every effort to find as many elite athletes after SCI from different continents as possible, analyzing a twenty-year period. The authors are aware that the number of subjects is a limitation, but this is the first international project related to the different aspects of the quality of life among people after SCI, as well as among elite athletes after SCI. We therefore hope that the results obtained in this project can be used as valuable and interesting material for further comparisons. Participants included int this study lived on different continents, which was why it was only possible to conduct this study remotely. The authors are also aware of the susceptibility to selection bias, as the number of participants was clearly lower than the number of people invited to the study (the project eventually included 8 participants out of 32 to whom the invitation was extended). There is therefore a likelihood of potential bias due to the fact that the subjects may have had more positive life experiences. However, the work aims to show that despite the traumatic injury, people who receive sufficient support and participate in sports find identity, belonging, and life satisfaction.

## 5. Conclusions

Despite the loss of fitness, elite athletes do not lose their identities. They live lives consistent with this identity, attempting to maintain it despite the loss of physical ability. Involvement in sports provides meaning and is a positive factor in the process of accepting disability as a necessary part of the adjustment process while providing group belonging. Most important is environments of people who can offer different perspectives or simply listen to them. It is therefore important for medical personnel to focus on not taking away hope as it is helpful for getting through the most difficult initial stage after the injury. There seems to be a particular role for the physiotherapists who work with such patients to help the patients look for ways in which they can realize and continue the lives they have led so far and to encourage them to, if possible, maintain or redefine particular identities. Since it is known what support effects are observed in elite athletes after SCI, it is advisable to apply appropriate support and strong promotion of participation in adapted sports, which would allow for expecting similar effects in the average patient population.

## Figures and Tables

**Table 1:** Sociodemographic data of study participants. C-cervical spine, Th–thoracic spine, L–lumbal spine.

Participant	Age	Gender	Nationality	Level of SCI	Years since Injury	Discipline before SCI	Sport after SCI
P1	41	Male	British	C3/4	14	BMX dirt jumps	No
P2	29	Male	Austrian	C6/7	5	Ski jumping	Rugby, skiing
P3	24	Female	Polish	Th11/12	6	Karate	Wheelchair dancing
P4	37	Male	British	C4/5	16	Rugby	No
P5	45	Female	Canadian	Th12/L1	14	Mountain Biking	Wheelchair basketball
P6	31	Male	British	Th6	15	Motocross	Car race
P7	40	Male	Polish	Th11	17	Judo	Canoe
P8	47	Male	Polish	L1/2	15	Speedway	Hand cycling

**Table 2:** Topics by subcategory.

Category	Subcategory	Quotation
Coping	Struggles	*P1: It is really difficult, your mental challenge is to keep going. Everything was a challenge when I woke up.*
	Fighting Spirit	*P7: I got into rehabilitation, intensive rehabilitation, which was replacing my sports training, and it lasted a couple of years.* *P8: “The sports anger in me woke up, I said to the doctor, you’ll see I’ll prove it to you I’ll come in on crutches, whatever.*
Athletic Identity	Fans’ support	*P1: I was very lucky because it was quite public. I was highlighted a lot from my crash. People in sports insurance were amazing, they have set up the fund, and people were donating money*
	The advantage of being an athlete	*P3: Karate before the accident taught me perseverance and diligence, which came in very handy later in this daily struggle.*
	Interaction with the sports community	*P2: The connection is still here and it’s always fun to watch them at their training, and it’s even more fun when I try to use their exercises for my training. And another thing is I just stand before coach exam, so I can train young ski jumpers. I wanted to stay here because i really lived in this area because of ski jumping and training. This is the middle point of my life.*
		*P4: with Leicester Tigers, I coach sometimes. I support beneficiaries for Leicester tigers charity. It’s pretty good really*
		*P5: and that gave me purpose and that was helpful for me but when that race ended and all through, but it gave me a purpose to get out of bed and to do something and so that was the that was sort of the baby steps.*
Adjustment	Finding the meaning of the accident	*P2: I stopped to complain about things which I cannot change. That I think, was one of the best things i learned because this save so much energy.* *P3: For sure life is reevaluating and I am so positive people admire me because I don’t worry about trivial things. P4: I believe, my accident happened for a reason so I can support young people who have a similar injury to me but don’t have that support I received.*
	Motivation sources	*P1: My kids, yeah. That’s the main thing that keeps me going.* *P1: After some time I was able to keep myself standing, it was so good for my mental condition, it was so inspiring and motivating.* *P2: When you see other people being proud of you because you just managed to move in the wheelchair.*
	Disability acceptance	*P5: I ended up I love the fact that I’m part of this community.*
	Goals, plans	*P1: I just want to do more motivational talks, raise my boys, they are great kids.* *P2: I want to raise the focus of the public when they build things to think about disable* *P4: The ambitions in my life is to make a foundation full of friends and spread the word about disability. I think as well we are changing the world for many disabled people, we empower them to do their best, make the most of their lives, try to look at the positives of their lives* *P6: at the minute my big goal is to compete the best I can with my disability in racing.*

## Data Availability

The datasets used and analyzed during this study are available from the corresponding author upon reasonable request.
